# A Comparative Study of Surgical and Endoscopic Gastrostomy Tube Insertion in Pediatric Patients

**DOI:** 10.7759/cureus.108783

**Published:** 2026-05-13

**Authors:** Talal Alshareef, Abdulmajeed Alenazi, Bilal Qayyum, Mohammad S Alonazi, Abdullah M Alotaibi, Mohammed Almohaidly, Mohamad Elmahmoud, Jamal Alhudaif, Abdullah Abduldaem, Mohanned Aljohany, Homoud Alhebbi, Faisal Alhaffaf, Sarah Algubaisi, Khalid A Albassam, Mohammed Alzahrani, Hawazin S Alqahtani

**Affiliations:** 1 General Surgery, Prince Sultan Military Medical City, Riyadh, SAU; 2 Pediatric Gastroenterology, Prince Sultan Military Medical City, Riyadh, SAU; 3 Pediatric Surgery, Prince Sultan Military Medical City, Riyadh, SAU; 4 Training, Saudi Commission For Health Specialties, Riyadh, SAU

**Keywords:** endoscopic gastronomy tube, gastrointestinal, gastronomy tube, intubation, pediatric patient, postoperative complications, retrospective studies, surgical gastronomy tube

## Abstract

Background: Gastrostomy tube (G-tube) insertion is a common procedure in pediatric patients requiring long-term enteral nutrition. While both surgical and endoscopic methods are widely used, comparative data on outcomes remain limited.

Objective: This study compares complication rates, hospital stay duration, recovery time, and reintervention rates between surgical and endoscopic G-tube insertion in pediatric patients.

Methods: A retrospective cohort study was conducted at Prince Sultan Military Medical City, Riyadh, Saudi Arabia, from 2020 to 2024, including 74 pediatric patients (35 surgical, 39 endoscopic). Data on demographics, procedural details, and postoperative outcomes were analyzed using appropriate statistical tests.

Results: No significant differences were found in baseline characteristics except for a higher prevalence of hypotonia/muscular dystrophy in the endoscopic group (15.38% vs. 0%, p=0.026). Postoperative weight improved significantly in the endoscopic group (p<0.001). Complication rates, including granuloma formation (14.29% vs. 25.64%, p=0.22), leakage (20% vs. 35.9%, p=0.13), and infection (2.86% vs. 10.26%, p=0.36), were comparable between surgical and endoscopic groups. Reintervention rates (5.71% vs. 5.13%, p>0.99) were similar.

Conclusion: Both surgical and endoscopic G-tube insertion techniques demonstrated comparable overall complication and reintervention rates in this cohort. Although the endoscopic approach was associated with improved postoperative nutritional recovery and earlier feeding resumption, most differences between groups did not reach statistical significance and should be interpreted cautiously. Further prospective multicenter studies with larger sample sizes are needed to optimize patient selection and procedural outcomes.

## Introduction

Gastrostomy tube (G-tube) insertion is an essential medical procedure for pediatric patients who require long-term enteral feeding due to various conditions, including neurological impairments, congenital anomalies, or chronic illnesses that limit oral intake [1,2]. This procedure provides adequate nutritional support and helps prevent malnutrition in children who are unable to feed orally. G-tube placement can be performed using two principal approaches: the traditional surgical method (open or laparoscopic), which is more invasive and requires general anesthesia and an abdominal incision, and the endoscopic method, which is minimally invasive and utilizes endoscopic guidance for tube insertion [3,4]. The choice of technique depends on multiple factors, including patient characteristics, physician expertise, and institutional resources. However, there remains limited consensus regarding the comparative outcomes of these approaches in pediatric populations [5].

Previous studies comparing surgical and endoscopic G-tube insertion have reported conflicting findings. Several studies demonstrated that endoscopic placement is associated with shorter recovery times and lower complication rates, whereas surgical placement may be associated with longer hospital stays and increased need for reintervention, but remains necessary in patients with anatomical or clinical contraindications to endoscopic placement [6-9]. Nevertheless, the existing pediatric literature is limited by small sample sizes, heterogeneous patient populations, and inconsistent reporting of postoperative and long-term outcomes, particularly in Middle Eastern populations [5,9,10].

In Saudi Arabia, the prevalence of pediatric conditions requiring long-term enteral feeding has increased, particularly among children with neurological impairments, congenital anomalies, and metabolic disorders [10]. Although minimally invasive gastrostomy techniques are increasingly utilized in tertiary pediatric centers, comparative data evaluating surgical versus endoscopic outcomes in Saudi pediatric populations remain scarce [11,12]. Consequently, regional evidence is needed to guide procedural selection and optimize patient outcomes.

Therefore, this study aims to compare complication rates, hospital stay duration, recovery time, and reintervention rates between surgical and endoscopic G-tube insertion in pediatric patients treated at a tertiary referral center in Saudi Arabia. Generating such evidence may help guide clinical decision-making and improve pediatric enteral feeding practices in similar healthcare settings.

## Materials and methods

This retrospective cohort study included all consecutive pediatric patients who underwent G-tube insertion at Prince Sultan Military Medical City, Riyadh, Saudi Arabia, between 2020 and 2024 in order to reduce selection bias. Patients were classified into surgical or endoscopic groups according to the gastrostomy insertion technique used. The decision regarding surgical versus endoscopic placement was based on multidisciplinary clinical evaluation, including patient anatomy, comorbid conditions, anesthetic risk, procedural feasibility, and treating physician preference, as no standardized institutional protocol for procedure selection was implemented during the study period. Procedures were performed or supervised by consultant pediatric surgeons and pediatric gastroenterologists experienced in G-tube placement at a tertiary referral center.

Patients who underwent G-tube insertion in combination with other procedures, such as fundoplication, as well as those who underwent alternative enteral access procedures, including jejunostomy, were excluded. Eligible patients were identified through institutional electronic medical records and operative/endoscopy registries. Cases with incomplete procedural documentation or combined procedures were excluded from the final analysis.

Data were retrospectively extracted from electronic medical records and included demographic characteristics (age, sex, weight, underlying diagnoses, and comorbidities), procedural details (type of anesthesia, insertion technique, and duration of procedure), and postoperative outcomes.

Outcomes

Outcomes were predefined prior to data extraction and categorized as primary and secondary outcomes.

Primary Outcomes

Hospital stay duration: This comprised the number of days from G-tube insertion to hospital discharge.

Recovery time: This included the number of days from insertion to successful resumption of enteral feeding, operationalized as tolerance of ≥50% of prescribed enteral feeds without vomiting, clinically significant gastric residuals, or cardiorespiratory instability [1,3].

Secondary Outcomes

Postoperative complications (within 30 days): These included bleeding, infection (local/systemic signs requiring antibiotics), tube dislodgement, peristomal leakage, mechanical obstruction, skin irritation, pain, accidental removal, sepsis/acute respiratory distress syndrome (ARDS), and peritonitis.

Reintervention rate: These comprised the need for additional procedures due to tube failure, complication, or conversion to an alternative tube type [2,4].

Nutritional response: This included a change in body weight from pre-procedure baseline to the first documented postoperative follow-up. 

Infection was defined as local or systemic signs of infection, including erythema, swelling, purulent discharge, or fever requiring antibiotic therapy [1,2]. Leakage was defined as persistent leakage of gastric contents around the gastrostomy site requiring medical or nursing intervention [1,4], whereas mechanical obstruction was defined as the inability to flush or utilize the tube requiring intervention or replacement [2].

Postoperative complications primarily represented early outcomes occurring within 30 days of the procedure unless otherwise documented during follow-up visits. Complications were identified retrospectively through review of electronic medical records, inpatient progress notes, procedural reports, and outpatient follow-up documentation. Outcome assessment was not blinded because of the retrospective nature of the study. Postoperative weight measurements were obtained from the earliest available follow-up assessment documented after G-tube placement.

Statistical analysis

All outcome definitions were established prior to data extraction to improve consistency, reproducibility, and comparability with previously published literature. Mortality was not evaluated as a comparative outcome because deaths, when present, were attributable to underlying disease processes rather than gastrostomy-related procedural complications. The study was conducted and reported in accordance with the Strengthening the Reporting of Observational Studies in Epidemiology (STROBE) guidelines for observational studies.

Continuous variables were assessed for normality using the Shapiro-Wilk test and are presented as mean ± standard deviation (SD) for normally distributed variables or median (interquartile range (IQR)) for non-normally distributed variables. Categorical variables are presented as frequencies and percentages. Comparisons between the surgical and endoscopic groups were performed using the independent samples t-test for normally distributed continuous variables and the Mann-Whitney U test for non-normally distributed variables. Categorical variables were analyzed using the chi-square (χ²) test or Fisher’s exact test, as appropriate. Paired comparisons were conducted using the Wilcoxon signed-rank test. Test statistics (e.g., t, χ², and U values) were reported alongside corresponding p-values. In addition to p-values, standardized effect sizes were considered to evaluate the magnitude of differences and baseline comparability between groups, recognizing that statistical significance alone may not adequately reflect clinical relevance in relatively small cohorts. A two-sided p-value <0.05 was considered statistically significant. Statistical analyses were performed using Stata 18.

Sample size estimation was based on previously published complication rates in pediatric gastrostomy studies. A systematic review and meta-analysis by Baker et al. demonstrated an approximate 15% to 20% difference in complication rates between surgical and endoscopic techniques. Assuming a conservative absolute difference of 15% (30% vs. 15%), with a two-sided alpha of 0.05 and 80% statistical power, the minimum required sample size was estimated to be 118 patients per group (total n ≈ 236). The present study included 74 patients, indicating limited statistical power to detect small-to-moderate differences between groups [5]. Therefore, non-significant findings should be interpreted cautiously, as the study may have been underpowered to detect clinically meaningful differences between techniques, thereby increasing the risk of type II error. Consequently, lack of statistical significance should not be interpreted as evidence of equivalence between the two approaches.

## Results

A total of 74 pediatric patients were included, with 35 undergoing surgical G-tube placement and 39 undergoing endoscopic G-tube placement. Overall, baseline demographic and clinical characteristics were comparable between groups, suggesting minimal selection bias. Although patients in the endoscopic group tended to be older (median seven vs. three years), this difference was not statistically significant (Mann-Whitney U = 530, p = 0.11). Similarly, there were no meaningful differences in sex distribution, anthropometric measures (weight and height), or geographic background (all p > 0.05), indicating that both groups were broadly similar at baseline.

Most underlying clinical conditions were also evenly distributed, including cerebral palsy, seizure disorders, developmental delay, gastroesophageal reflux, and cardio-respiratory disease, none of which differed significantly between groups. However, a notable exception was the higher prevalence of hypotonia/muscle dystrophy in the endoscopic group (15.38% vs. 0%; Fisher’s exact p = 0.02). This finding suggests a potential selection pattern in which patients with neuromuscular disorders were more likely to undergo endoscopic placement, possibly due to perceived lower procedural invasiveness or surgical risk. Other less common comorbidities, including metabolic disorders and congenital anomalies, were rare and did not differ between groups. Preoperative nutritional status, as reflected by serum albumin levels, was similar between groups (38.2 ± 5.2 vs. 40.3 ± 4.7 mg/dL; t (72) = −1.80, p = 0.07), further supporting baseline comparability (Table 1).

**Table 1:** Baseline data comparing patients with surgical and endoscopic gastrostomy tube placement. Continuous variables are presented as mean ± standard deviation or median (interquartile range) based on distribution. Categorical variables are presented as frequencies (%). Statistical tests were selected based on data type and distribution.

Variables	Surgical (n= 35)	Endoscopic (n= 39)	P-value	Test statistics
Age (years)	3 (2- 7)	7 (3- 10)	0.11	Mann-Whitney U= 530
Male sex, n (%)	17 (51.52%)	22 (56.41%)	0.67	Chi-square χ²(df) = 0.17
Weight (kg)	12.15 (7.9- 15.1)	10.85 (7.2- 20)	0.92	Mann-Whitney U= 673
Height (cm)	93± 28	101± 28	0.25	t (72) = -1.15
Residency region: Central	28 (84.85%)	31 (79.49%)		
Residency region: Eastern	1 (3.03%)	2 (5.13%)		
Residency region: Northern	2 (6.06%)	1 (2.56%)		
Residency region: Southern	2 (6.06%)	3 (7.69%)		
Residency region: Western	0	1 (2.56%)		
Non-national	0	1 (2.56%)		
Cerebral palsy	3 (8.57%)	9 (23.08%)	0.12	χ² (1) =2.42
Seizure	14 (40%)	11 (28.21%)	0.28	χ² (1) =1.15
Global developmental delay	12 (34.29%)	15 (38.46%)	0.70	χ² (1) =0.14
Hypotonia/muscle dystrophy	0	6 (15.38%)	0.02	
Hypoxic-ischemic encephalopathy	0	2 (5.13%)	0.49	
Apert syndrome	0	1 (2.56%)	>0.99	
Cleft lip/palate	4 (11.43%)	2 (5.13%)	0.41	
Choanal atresia	0	1 (2.56%)	>0.99	
Esophageal atresia/ trachea-esophageal fistula	1 (2.86%)	1 (2.56%)	>0.99	
Laryngomalacia/ tracheomalacia	2 (5.71%)	2 (5.13%)	>0.99	
Pyloric/duodenal atresia	0	1 (2.56%)	>0.99	
Gastro-esophageal reflux	13 (37.14%)	18 (46.15%)	0.43	χ² (1) =0.61
Gastrointestinal dysmotility	2 (5.71%)	3 (7.69%)	>0.99	
Metabolic disorders	1 (2.86%)	4 (10.26%)	0.36	
Infantile intractable diarrhea	1 (2.86%)	0	0.47	
ADHD	1 (2.86%)	0	0.47	
Swallowing dysfunction	5 (14.29%)	9 (23.08%)	0.33	
Cardio-respiratory disease	12 (34.29%)	20 (51.28%)	0.14	χ² (1) =2.17
Albumin (mg/dl)	38.2± 5.2	40.3± 4.7	0.07	t (72) = -1.80

Overall, postoperative weight improved significantly after treatment based on Wilcoxon signed-rank analysis (Z = −4.92, p < 0.001). When analyzed by group, the improvement in weight was not statistically significant in the surgical group (p=0.06) but was significant in the endoscopic group (p<0.001).

Postoperative complications were broadly comparable between the surgical and endoscopic groups, with no statistically significant differences observed across outcomes. Although rates of local tube-related complications such as granuloma formation and leakage were numerically higher in the endoscopic group (25.64% vs. 14.29% and 35.90% vs. 20%, respectively), these differences did not reach statistical significance (χ² (1) = 1.47, p = 0.22; χ² (1) = 2.29, p = 0.13), suggesting similar safety profiles between techniques. Other minor complications, including skin irritation and mechanical obstruction, occurred at comparable rates and were clinically infrequent.

Infectious complications were uncommon overall. While infection appeared more frequent in the endoscopic group (10.26% vs. 2.86%), this difference was not statistically significant (Fisher’s exact p = 0.36). Similarly, rare but clinically relevant complications such as accidental tube removal, bleeding, and sepsis/ARDS were observed at very low and comparable rates. Reintervention rates were low and similar in both groups, with approximately 5% of patients requiring tube replacement (p > 0.99). Procedure-specific adjustments were infrequent, with one surgical patient requiring conversion to percutaneous endoscopic gastrostomy (PEG) and two endoscopic patients requiring conversion to a Mic-Key G-tube (Table 2).

**Table 2:** Comparison of postoperative complications and outcomes between both groups Continuous variables are presented as mean ± standard deviation or median (interquartile range) based on distribution. Categorical variables are presented as frequencies (%). Statistical tests were selected based on data type and distribution. Complication rates represent postoperative events occurring within 30 days following gastrostomy tube insertion.

Variables	Surgical (N= 35)	Endoscopic (N= 39)	P-value	Test statistics
Granuloma	5 (14.29%)	10 (25.64%)	0.22	Chi-square χ² (1) =1.47
Leak	7 (20%)	14 (35.90%)	0.13	χ² (1) =2.29
Skin irritation	2 (5.71%)	4 (10.26%)	0.67	
Infection	1 (2.86%)	4 (10.26%)	0.36	
Mechanical obstruction	4 (11.43%)	4 (10.26%)	>0.99	χ² (1) =0.02
Pain	0	2 (5.13%)	0.49	
Accidental removal	1 (2.86%)	1 (2.56%)	>0.99	χ² (1) =0.001
Sepsis/Acute respiratory distress syndrome (ARDS)	1 (2.86%)	0	0.47	
Peritonitis	2 (5.7%)	0	0.23	
Bleeding	1 (2.86%)	1 (2.56%)	>0.99	χ² (1) =0.001
Change to percutaneous endoscopic gastrostomy (PEG)	1 (2.86%)	-		
Change to Mic-Key tube	-	2 (5.13%)		
Tube replacement	2 (5.71%)	2 (5.13%)	>0.99	χ² (1) =0.01
Hospital stay (days)	5	3	0.06	Mann-Whitney U= 462
Recovery time (days)	2	1	0.03	Mann-Whitney U= 389

## Discussion

Our comparative analysis of surgical versus endoscopic G-tube insertion in pediatric patients provides important insights into the relative safety and effectiveness of these approaches. The two groups were well matched in baseline demographic and clinical characteristics, minimizing confounding and strengthening the validity of outcome comparisons. Although the age difference between groups did not reach statistical significance, patients in the endoscopic group tended to be older, which may still carry clinical relevance given potential differences in feeding tolerance, comorbidity profiles, and postoperative recovery patterns across pediatric age groups. This comparability supports the reliability of observed differences in postoperative recovery and complications.

Consistent with existing literature, our findings suggest that endoscopic G-tube placement is associated with favorable clinical outcomes. Previous evidence, including the meta-analysis by Baker et al., has demonstrated that PEG is linked to reduced postoperative pain, shorter procedural duration, and faster recovery compared to surgical techniques [13]. Although numerically fewer major complications were observed in the endoscopic group, these differences did not reach statistical significance and should therefore be interpreted cautiously. This likely reflects the minimally invasive nature of the procedure, which avoids abdominal incision and reduces tissue trauma.

Our findings also align with the prospective cohort study by Lautz et al., which reported shorter hospital stays and lower readmission rates in patients undergoing endoscopic placement [14]. A shorter median hospital stay was observed in the endoscopic group (five days vs three days), although this difference did not reach statistical significance. More importantly, endoscopic patients resumed feeding earlier (median one day vs. two days), a finding that is both statistically significant and clinically relevant. Early initiation of enteral feeding is a critical outcome in pediatric populations, as it directly impacts nutritional recovery and overall morbidity.

In contrast to some prior studies, such as the analysis by Sandberg et al., which reported no difference in major complications between techniques [15], our data showed a numerically higher rate of severe complications in the surgical group, including cases of peritonitis. Although these events were rare, they highlight the potential risks associated with more invasive approaches. Differences in institutional protocols, surgical expertise, and postoperative care may explain these variations across studies.

An important observation in our study was the higher prevalence of hypotonia and neuromuscular disorders in the endoscopic group. This likely reflects an important source of selection bias, whereby clinicians may have preferentially selected the less invasive endoscopic approach for medically complex or higher-risk patients. Such non-random group allocation may have influenced postoperative outcome comparisons and should be considered when interpreting the findings. This is supported by findings from McSweeney et al., who noted that neurologically impaired children often present unique challenges and higher complication risks regardless of technique [16]. Despite this, the comparable outcomes observed in our study further support the safety of endoscopic placement even in vulnerable populations.

Previous studies have reported shorter operative times with percutaneous endoscopic gastrostomy compared with surgical gastrostomy techniques [17]. Although operative time was not systematically recorded in the present study and therefore could not be formally compared, the observed postoperative recovery trends in our cohort were generally consistent with the minimally invasive advantages described in prior literature.

Our findings are further supported by multicenter data from Petrosyan et al., which demonstrated earlier feeding initiation and reduced postoperative ileus with endoscopic placement [17]. The consistency of these findings across different settings reinforces the clinical advantage of the endoscopic approach in promoting faster recovery and nutritional rehabilitation. Additionally, mortality was not included as a comparative outcome measure in this study, as deaths were related to patients’ underlying primary diseases and comorbid conditions rather than technical or procedural factors associated with G-tube insertion.

Several limitations should be considered when interpreting our results. First, the retrospective design introduces inherent risks of selection bias and unmeasured confounding. Accordingly, causal relationships between gastrostomy technique and clinical outcomes cannot be definitively established. Second, the relatively small sample size may limit statistical power, particularly for detecting differences in rare complications. Third, the single-center nature of the study may affect generalizability to other institutions with differing expertise and protocols. Additionally, long-term outcomes such as tube longevity, quality of life, and late complications were not assessed. Finally, the absence of standardized criteria for procedure selection may have influenced group allocation and outcomes. The study may have been underpowered to detect small-to-moderate differences in major complications and hospital stay outcomes, increasing the possibility of type II error.

Despite these limitations, this study adds valuable data from a Saudi pediatric population and supports the growing evidence favoring endoscopic gastrostomy as a safe, minimally invasive, and clinically effective option. Future prospective multicenter studies with larger cohorts and standardized protocols are needed to confirm these findings and optimize patient selection strategies.

## Conclusions

Our findings suggest that endoscopic G-tube placement is a safe and effective alternative to surgical gastrostomy in pediatric patients. Both techniques demonstrated comparable complication and reintervention rates, indicating similar overall safety profiles in this cohort. Although the endoscopic approach was associated with earlier resumption of feeding and a trend toward shorter hospital stay, these findings should be interpreted cautiously, as most differences were not statistically significant. The minimally invasive nature of endoscopic placement may offer potential advantages in postoperative recovery, particularly in medically complex patients; however, the retrospective design, relatively small sample size, and potential selection bias limit definitive conclusions regarding superiority between techniques. Further prospective, multicenter studies with larger cohorts and longer follow-up are required to validate these findings and better define optimal patient selection and long-term outcomes.
